# Functional characterization of common BCL11B gene desert variants suggests a lymphocyte-mediated association of BCL11B with aortic stiffness

**DOI:** 10.1038/s41431-018-0226-z

**Published:** 2018-08-08

**Authors:** Raya Al Maskari, Iris Hardege, Sarah Cleary, Nicki Figg, Ye Li, Keith Siew, Ashraf Khir, Yong Yu, Pentao Liu, Ian Wilkinson, Kevin O’Shaughnessy, Yasmin

**Affiliations:** 1 Division of Experimental Medicine & Immunotherapeutics (EMIT), Department of Medicine, University of Cambridge, Cambridge, UK; 2 Division of Cardiovascular Medicine, Department of Medicine, University of Cambridge, Cambridge, UK; 3 Brunel Institute of Bioengineering, Brunel University, Middlesex, UK; 4 Wellcome Trust Sanger Institute, Hinxton, Cambridge UK

## Abstract

The recent genome-wide analysis of carotid–femoral pulse wave velocity (PWV) identified a significant locus within the 14q32.2 gene desert. Gene regulatory elements for the transcriptional regulator B-cell CLL/lymphoma 11B (BCL11B) are within this locus and an attractive target for the gene association. We investigated the functional impact of these gene desert SNPs on BCL11B transcript in human aorta to characterize further its role in aortic stiffness. To do this, we used a large repository of aortic tissues (*n* = 185) from an organ transplant program and assessed ex vivo stiffness of the aortic rings. We tested association of three lead SNPs from the GWAS meta-analysis with ex vivo aortic stiffness and BCL11B aortic mRNA expression: rs1381289 and rs10782490 SNPs associated significantly with PWV and showed allele-specific differences in BCL11B mRNA. The risk alleles associated with lower BCL11B expression, suggesting a protective role for BCL11B. Despite strong association, we could not detect BCL11B protein in the human aorta. However, qPCR for CD markers showed that BCL11B transcript correlated strongly with markers for activated lymphocytes. Our data confirm the significance of the 14q32.2 region as a risk locus for aortic stiffness and an upstream regulator of BCL11B. The BCL11B transcript detected in the human aorta may reflect lymphocyte infiltration, suggesting that immune mechanisms contribute to the observed association of BCL11B with aortic stiffness.

## Introduction

Aortic stiffness (AS) underlies systolic hypertension, promotes heart failure and is associated with increased cardiovascular (CV) morbidity and mortality [1]. AS strongly correlates with left ventricular hypertrophy and aortic aneurysms [2, 3] and is linked to the pathogenesis of cognitive impairment, stroke and renal failure [3]. Carotid–femoral pulse wave velocity (cfPWV), the current gold-standard measure of AS, is an independent predictor of CV events and all-cause mortality in both healthy individuals and high-risk groups, including end-stage kidney disease, hypertension, and diabetes [4].

As with most other CV traits, AS is genetically complex and moderately heritable, but precise molecular mechanisms are still lacking. The most recent genome-wide analysis of cfPWV in the *AortaGen* Consortium identified a significant locus on chromosome 14 [5]. The top signals from this genome-wide association study (GWAS) meta-analysis reside in a gene-poor region (14q32.2), although the region contains regulatory elements that may influence AS by regulating gene expression in a *trans* and tissue-specific manner. In fact, the cfPWV locus maps to an enhancer for B-cell chronic lymphocytic leukemia/lymphoma 11B (BCL11B), which resides ~ 850 kb upstream from the locus [6]. BCL11B, also known as chicken ovalbumin upstream promoter transcription factor (COUP-TF) interacting protein 2 (CTIP2), is a typical transcriptional regulator with six C_2_H_2_-like zinc finger domains. It is an attractive AS candidate for several reasons. It is highly expressed and crucial to cutaneous, immune, and central nervous system function and is a key regulator of adipogenesis [7–9]. BCL11B also governs crucial aspects of the development, differentiation, recruitment, and function of T lymphocytes and other immune cells [10]. Significantly, for blood vessels, the interaction of BCL11B with the COUP-TFII nuclear receptor regulates arterial–venous blood vessel identity [11, 12]. Additionally, altering the expression of BCL11B perturbs several genes and signaling pathways that have recognized roles in vascular calcification and extracellular matrix synthesis, deposition, turnover, and assembly [9, 13, 14]. Clinical support also comes from the recent Twins UK Cohort, where BCL11B transcript levels in circulating lymphocytes were correlated with cfPWV and decreased carotid distensibility [15].

Despite the accumulating evidence implicating BCL11B as a novel candidate gene for AS, its exact molecular role remains unclear. Our working hypothesis has been that it may regulate the expression of genes that either directly regulate the architecture of the vessel wall or promote vascular calcification and/or inflammation. Hence, we undertook this study to examine the functional impact of the lead single-nucleotide polymorphisms (SNPs) from the *AortaGen* Consortium on aortic BCL11B transcript levels and on ex vivo measurements of AS in a large sample of human aortic tissues as well as to determine the expression pattern of BCL11B mRNA in the human aorta.

## Materials and methods

### Study samples

Aortic tissue samples were harvested from organ donors through transplant coordinators at Addenbrooke’s Hospital, Cambridge. Fresh tissue from the ascending, arch, thoracic, and abdominal segments of the aorta and iliac arteries were preserved in tissue medium at the time of organ donation. Tissues were cleaned of adherent blood vessels or adipose tissues and then stored at −80 °C. Two-cm rings were cut for biophysical measurements and sections were preserved in RNA*later*^®^ for RNA extraction.

Demographic data, anthropometric information, biochemical and hemodynamic measurements, medical and drug history (past and at the time of death) where available, cause of death, and other details that may have affected AS were recorded. All samples and donor data were handled in accordance with the policies and procedures of the Human Tissue Act (UK). The Local and Regional Ethics Committees approved the study (MREC/03/2/074).

### Biophysical measurements

Aortic ring diameter and wall thickness were recorded with digital calipers, and Young’s modulus (EM) was measured using an Instron 5542 tensile test machine controlled by the Bluehill 2 software as detailed elsewhere [16]. Briefly, each ring was cycled 5 times in the range of 0–200 mmHg at a rate of 10 mm/min. EM at a load of 100 mm Hg was used to calculate pulse wave velocity (PWV^MK^) for each aorta via the Moens–Kortweg equation: $\sqrt {{\mathrm{EM}} \times \frac{h}{{2r{\mathrm{\rho }}}}}$ where *h* is wall thickness, *r* is the arterial radius, and *ρ* is blood density (taken as 1.05 g/cm^3^) [16].

### DNA extraction and SNP genotyping

Genomic DNA was extracted from thawed aortic tissue following the manufacturer’s guidelines of the QIAmp DNA Mini Kit (QIAGEN). DNA quantity and purity were checked on a NanoDrop spectrophotometer ND100. The three SNPs used (IDs from build 151 of dbSNP) were: rs1381289 (ch14:g.98126027C>T), rs10782490 (ch14:g.98083046C>T), and rs17773233 (ch14:g.98116322G>T) and genotyped using TaqMan SNP Genotyping assays (ThermoFisher Scientific). They were the top SNPs in the previous *AortoGen* meta-analysis and are in tight linkage disequilibrium (*D*’ = 1; rs1381289 vs rs10782490 *R*^2^ = 0.78, rs1381289 vs rs17773233 *R*^2^ = 0.42, rs10782490 vs rs17773233 *R*^2^ = 0.32). Allelic discrimination was carried out using the ABI 7500 Detection System (ThermoFisher Scientific). The genotyping data for the three SNPs together with the corresponding phenotypic data from our donor cohort has been deposited publically on the Leiden Open Variant Database (http://www.lovd.nl/3.0/home) at https://databases.lovd.nl/shared/variants/0000368472. The summarized phenotypic data by genotype is also included as a table in Supplementary File.

### RNA extraction and cDNA preparation

Total RNA was extracted from aortic tissue preserved in RNAlater® Stabilization Solution (ThermoFisher Scientific) using the RNeasy Fibrous Tissue Mini Kit (QIAGEN) following the manufacturer’s guidelines. Peripheral blood mononuclear cell (PBMC) total RNA was extracted using the PaxgeneTM Blood RNA Validation Kit (PreAnalytix). RNA quantity and purity were determined using the NanoDrop ND100 spectrophotometer. Complementary DNA (cDNA) was generated using the GoScriptTM Reverse Transcription System (Promega), and cDNA samples were stored at −20 °C until analyzed.

### Gene expression

Gene expression levels of BCL11B, CD45, CD25, CD8α, and CD235α were quantified using TaqMan^®^ probe and primers (assay IDs: BCL11B Hs01102259_m1 human; CD45 PTPRC Hs 04189704_m1; CD25 IL2RA Hs 00907778_m1; CD8α Hs00233520_m1; CD235α Hs01068072_m1, ThermoFisher Scientific). Human GAPDH endogenous control (assay ID: 4326317E, ThermoFisher Scientific) was used to normalize the data. Real-time PCR was performed in duplicate using the TaqMan^®^ Fast Advanced Master Mix (ThermoFisher Scientific) using 1.5 μl stock cDNA template. Gene expression data are represented as 2^−^^ΔCt^ where ΔCt = GAPDH Ct−target gene Ct [17].

### Western blotting

Frozen aortic tissues were homogenised using TissueLyser LT (QIAGEN) and protein lysates were extracted in NE-PER nuclear and cytoplasmic extraction buffers (ThermoFisher Scientific) containing protease inhibitors (Calbiochem). Full-length CTIP2 expressed in a pcDNA3.1 vector and transfected into HEK293T cells was used as a positive control for the antibody and empty vector transfected into HEK293T cells was used as a negative control. Cells were lysed using RIPA buffer and protein concentrations were determined with the PierceTM BCA protein assay (ThermoFisher Scientific). Protein lysates (10 μg) were incubated in a Laurel DuodecylSulfate sample loading buffer and Bolt® sample reducing agent (ThermoFisher Scientific) before loading and separation by sodium dodecyl sulfate-gel electrophoresis in 4–12% gradient Bis-Tris Plus Bolt® gels (ThermoFisher Scientific) at 200 V for 30 min. They were transferred to 0.22 μM nitrocellulose membrane (ThermoFisher Scientific) using the iBlot2 dry blotting system (ThermoFisher Scientific) and blocked with 5% (wt/vol) milk in TBS buffer and then incubated with primary antibodies in TBS-Tween (0.1% Tween 20) for 16 h at 4 °C. Secondary antibodies were incubated in TBS-Tween for 1 h at room temperature (RT) in the dark. Membranes were washed in TBS-Tween 3× for 15 min between primary and secondary antibody incubations and visualized using the LI-COR Odyssey system.

### Immunohistochemical (IHC) staining

IHC staining was performed in formalin-fixed, paraffin-embedded (FFPE) aortic samples. Human tonsil sections were used as a positive control. Briefly, 4 µm sections were deparaffinized in Histoclear (National Diagnostics) and then dehydrated through graduated alcohols. Antigen retrieval was performed in R-Universal epitope recovery buffer (Aptum Biologics Ltd #AP0530-125) using 2100 Retriever (Aptum Biologics Ltd). EnVision^TM^+Dual Link system (Dako) was used for chromogenic detection of the primary antibodies. Sections were counterstained with hematoxylin (Sigma-Aldrich), rehydrated through graduated alcohols, cleared in Histoclear, and then mounted with Histomount (National Diagnostics).

### Immunofluorescence staining with tyramide signal amplification (TSA)

TSA staining was performed on three FFPE aortic sections that showed highest BCL11B mRNA and tonsil sections were used as a positive control. As TSA is catalyzed by peroxidase, endogenous peroxidase activity was quenched with 3% H_2_O_2_ in methanol for 30 min. Sections were blocked in TNB buffer made with 0.1 M Tris-HCl (pH 7.5), 0.15 M NaCl and 0.5% Blocking Reagent (FP1012, PerkinElmer) for 1 h at RT followed by primary antibody incubation in TNB buffer for 1 h at RT. Sections were washed in TNT wash buffer (0.1 M Tris-HCl, 0.15 M NaCl, and 0.05% Tween-20) and then incubated in horseradish peroxidase-conjugated secondary antibody (Sigma-Aldrich) diluted at 1:1000. Sections were incubated in 200 μl TSA Plus Fluorescein working solution (PerkinElmer) for 5 min and washed in TNT buffer. Sections were counterstained with Sytox® Orange (ThermoFisher Scientific), mounted with ProLong® Gold, and cured overnight before visualization with Leica SP8 confocal laser scanning microscope.

### Antibodies

BCL11B/CTIP2 expression in the aorta was probed using five antibodies that recognize different epitopes on CTIP2: anti-CTIP2 rat monoclonal (Abcam, ab18465), rabbit polyclonal (Abcam, ab70452), rabbit polyclonal (Abcam, ab28448), rabbit monoclonal (Abcam, ab187668), and rabbit monoclonal (CST signaling 12120). Western blot and IHC staining antibody dilutions were used as recommended by the manufacturer. CD45 expression in the aorta was probed with anti-CD45 rabbit polyclonal (Abcam, ab10558) at 1:2000 dilution. Beta-actin rabbit polyclonal immunoglobulin G (ThermoFisher Scientific) was used for the western blot loading control at 1:1000 dilution. Donkey anti-rabbit (LI-COR) IRDye® 800CW and goat anti-rat (ThermoFisher Scientific) Alexa Fluor® 680-conjugated secondary antibodies were used at 1:5000 dilution for western blot.

### BCL11B expression in mice

Brain, thymus, spleen and whole aortic tissues were harvested from 10-week-old wild-type mice (*n* = 9) and preserved in either RNAlater® for gene expression and western blotting and then stored at −80 °C or in 4% (vol/vol) paraformaldehyde overnight for IHC staining. The aortae were perfused with RNA-free phosphate-buffered saline three times immediately after harvesting to remove blood and adipose tissue was trimmed from aortic samples before western blotting and gene expression. RNA was extracted using the RNeasy Mini Kit (QIAGEN) and cDNA was generated using GoScriptTM Reverse Transcription (Promega) as described above. BCL11B mRNA was probed using TaqMan® gene expression assay (Mm00480516_m1) and Eukaryotic 18S rRNA Endogenous Control (ThermoFisher Scientific) for normalization. Protein was extracted from tissues using RIPA buffer supplemented with protease inhibitors (Calbiochem). BCL11B was probed using rabbit polyclonal anti-BCL11B (Abcam, ab70452) at 1:200 dilution for western blotting as described earlier. The EnVisionTM+Dual Link system (Dako) was used for IHC detection with rabbit polyclonal anti-BCL11B (Abcam, ab70452) at 1:1000 dilution.

BCL11B was further probed in tissues harvested from 10-week-old BCL11B-tdTomato reporter mice [16]. The reporter mice were a C57BL/6 background and the tdTomato cassette (loxP-IRES-dtTomato) was targeted to the 3’ untranslated region of BCL11B. To generate conditional knockouts, mice were injected with tamoxifen 1 week prior to tissue harvesting resulting in deletion of exon 4 of BCL11B. Rabbit polyclonal anti-RFP (Rockland Immunochemicals, 600–401–379) was used at 1:2000 dilution for western blot detection. Human colorectal cells expressing tdTomato (generous gift from Fatima Junaid, Cancer Research UK, Cambridge) were used as  positive control.

### Statistical analysis

Statistical analyses were conducted using the SPSS version 23 and GraphPad Prism 7 software. As the thoracic section of the aorta differs histologically and embryologically from the abdominal segment, statistical analysis used samples from the ascending aorta, arch and thoracic aorta only (*n* = 185 of the 209 aortae collected). Deviations from the Hardy–Weinberg equilibrium were tested using χ^2^ test. For each SNP, *t* tests were used to compare BCL11B mRNA differences between the two homozygous allele carriers. Since SNP genotype showed a dose-dependent pattern of inheritance on the phenotype (PWV and BCL11B mRNA), SNP associations with PWV were tested assuming a standard additive model using regression models adjusted for factors that influence PWV (age, age^2^, gender, height, and weight). Spearman’s correlation coefficients were used to determine correlations between BCL11B and CD markers. In all statistical tests, a *P* value <0.05 was considered significant.

## Results

The clinical and demographic features of the donor samples are provided in Table 1. Less than 10% had established CV disease or were diabetic. However, a third of the aortic donors were hypertensive.

**Table 1:** Baseline characteristics of the study cohort

Parameters	Donor features (*N* = 209)	Range (min.–max.)
Male/female (*n*)	122/87	—
Age (years)	57 (19)	17–83
Height (m)	1.7 (0.2)	1.45–2.0
Weight (Kg)	80 (20)	47–160
BMI (Kg/m^2^)	27.04 (6)	16–69
SBP (mm Hg)	120 (32)	40–260
DBP (mm Hg)	68 (14)	20–120
Elastic modulus (MPa)	0.14 (0.09)	0.05–0.63
PWV^MK^ (m/s)	3.53 (0.97)	2.4–9.49
CVD (*n*)	21	—
HT on treatment (*n*)	62	—
Diabetes (*n*)	16	—
Hyperlipidemia (*n*)	9	—

Data are median (IQR)

*BMI* body mass index, *SBP* systolic blood pressure, *DBP* diastolic blood pressure, *PWV*^*MK*^ pulse wave velocity calculated using Moens–Kortweg equation, *CVD* cardiovascular disease, *HT* hypertension

### Association of 3’BCL11B gene desert SNPs with AS

We investigated the association of AS measured ex vivo in donor aortae with the lead SNPs from the *AortaGen* Consortium GWAS meta-analysis. Hardy–Weinberg equilibrium was satisfied for all the SNPs tested (Table 2). Allele frequencies of the SNPs in our aortic tissue resource were comparable to those observed in the GWAS meta-analysis. The minor allele frequencies were 0.45 vs 0.44 for rs1381289, 0.50 vs 0.47 for rs10782490, and 0.19 vs 0.19 for rs17773233 (donor aortae vs *AortaGen* Consortium).

**Table 2:** Chromosome 14 polymorphisms with genotype and allele frequencies

SNPs	Genotypes	*N* (%)	Hardy–Weinberg test	Alleles	*N* (%)
rs1381289 (C>T)	CC	60 (29.1)	*χ*^2^ = 0.312	C	226 (54.9)
	CT	106 (51.5)	*P* = 0.576	T	186 (45.1)
	TT	40 (19.4)			
rs10782490 (C>T)	CC	49 (23.9)	*χ*^2^ = 0.395	C	205 (50.0)
	CT	107 (52.2)	*P* = 0.530	T	205 (50.0)
	TT	49 (23.9)			
rs17773233 (G>T)	GG	138 (66.4)	*χ*^2^ = 0.098	G	338 (81.2)
	GT	62 (29.8)	*P* = 0.754	T	78 (18.8)
	TT	8 (3.8)			

**Fig. 1:**
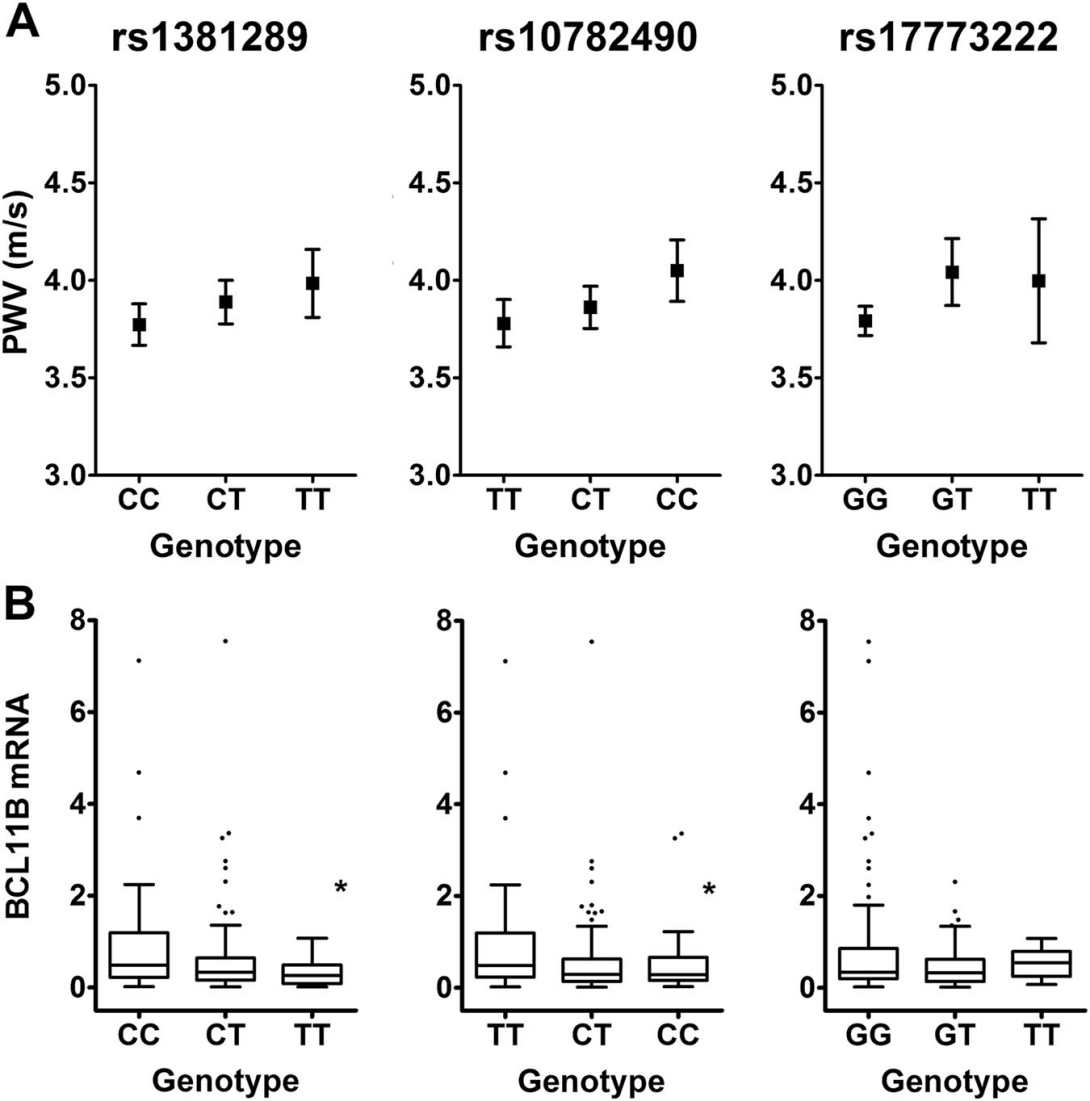
Association of 3’BCL11B gene desert SNPs with (**a**) calculated PWV and (**b**) BCL11B mRNA in human aortic tissues. Boxes are interquartile ranges, the lines within boxes are median values and whiskers are 5th and 95th percentiles. *P < 0.05 for comparison of the two homozygous genotypes

All three SNPs from the GWAS meta-analysis (rs1381289, rs10782490 and rs17773233) showed an allele–dose trend with calculated PWV (PWV^MK^, Fig. 1). Multiple linear regression models including traditional confounders for PWV (age, age^2^, gender, height, and weight) showed that two SNPs (rs1381289 and rs10782490) associated significantly and independently with PWV (Table 3).

**Table 3:** Multiple regression analysis showing parameters associated with inverse PWV

Model parameters	Standardized coefficients (beta)	*t*	Significance level (*P*)
Dependent variable: inverse PWV			
Age	1.967	5.993	<0.001
Age^2^	−2.495	−7.708	<0.001
Gender	−0.009	−0.127	0.899
Height	0.063	0.773	0.441
Weight	−0.049	−0.765	0.445
**rs1381289 C**>**T**	−0.157	−2.746	**0.007**
Adjusted *R*^2^ = 0.446; *F* = 24.074; *P* < 0.001			
Age	1.877	5.627	<0.001
Age^2^	−2.409	−7.273	<0.001
Gender	0.003	0.043	0.966
Height	0.073	0.892	0.374
Weight	−0.053	−0.814	0.417
**rs10782490 C**>**T**	−0.120	−2.078	**0.039**
Adjusted *R*^2^ = 0.432; *F* = 22.663; *P* < 0.001			
Age	1.900	5.726	<0.001
Age2	−2.428	−7.408	<0.001
Gender	0.023	0.318	0.751
Height	0.079	0.971	0.333
Weight	−0.047	−0.728	0.468
**rs17773233 G**>**T**	−0.098	−1.706	**0.090**
Adjusted *R*^2^ = 0.429; *F* = 22.766; *P* < 0.001			

### 3’BCL11B gene desert SNPs and aortic BCL11B gene expression

Because the SNPs investigated lie within a region harboring gene regulatory elements, we looked for evidence that these variants had distal regulatory effects on BCL11B transcript, the nearest known gene (upstream) of this locus on the minus strand. We estimated allelic effects by comparing the mean expression of BCL11B mRNA in the two homozygote classes for each SNP (Fig. 1b). The homozygous CC of the lead SNP rs1381289 showed three-fold higher mean BCL11B expression compared with the homozygous T allele (*P* = 0.0005). Heterozygous individuals displayed an intermediate phenotype. Consistent with this, the homozygous risk allele (CC) of rs10782490 also showed lower BCL11B transcript compared with the homozygous T allele (1.8-fold, *P* = 0.05). However, BCL11B transcript levels were not significantly influenced by rs17773233 genotype, although there was a trend toward lower BCL11B mRNA among carriers of the risk allele. Together, this data demonstrates that two of the risk SNPs are associated with lower BCL11B expression and behave as expression-SNPs (e-SNPs). We further examined the expression pattern of BCL11B along the length of the aorta and found that BCL11B transcript was most abundant in the ascending aorta, followed by the thoracic, abdominal aorta, and iliac arteries (Supplementary Fig. 1).

### No evidence of BCL11B/CTIP2 protein expression in the human aorta

**Fig. 2:**
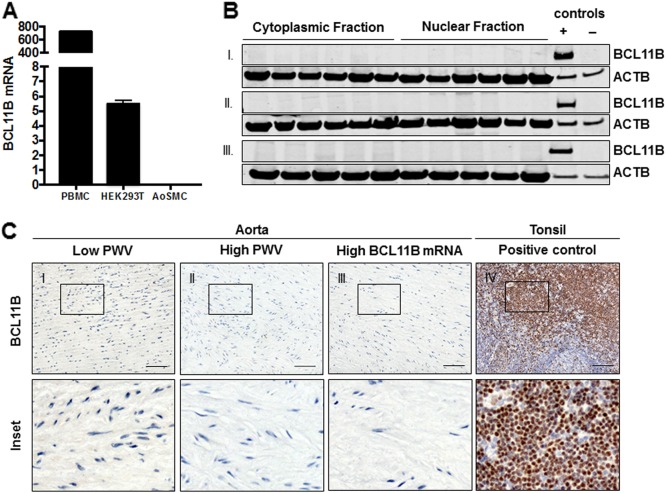
Aortic expression of BCL11B. (**a**) Bar chart showing reduction in BCL11B mRNA expression in cultured human aortic vascular smooth muscle cells is insignificant compared to PBMC and HEK293T cells. (**b**) Representative western blot of aortic BCL11B shows negative staining across nuclear and cytoplasmic homogenates of (I) low PWV samples, (II) high PWV samples, and (III) samples with the highest BCL11B mRNA levels. Positive control is protein lysate of HEK293T-expressing full-length BCL11B and negative control is protein lysate of HEK293T expressing an empty vector control. (**c**) Representative immunohistochemical staining of (*n* = 45) aortic samples shows lack of BCL11B expression in the aorta. Tonsil was used as a positive control for the antibody (scale bar = 50 μM)

To examine the functional significance of BCL11B in the adult aorta, we probed its expression using antibodies that target different epitopes on the protein. We selected samples from the extreme ends of the PWV spectrum (low PWV and high PWV, *n* = 15 in each group) as well as additional (*n* = 15) samples that had the highest BCL11B gene expression. We did not detect CTIP2 in any of these samples using either western blotting of aortic homogenates or IHC staining of FFPE sections (Fig. 2a–c). We also used TAS to increase the detection sensitivity of the IHC but still failed to identify BCL11B in the human aorta (Supplementary Fig. 2). This led us to question whether BCL11B is constitutively expressed in aortic smooth muscle cells (AoSMCs) or whether the levels detected in the aortic tissue samples reflected underlying infiltration from circulating lymphocytes. In fact, BCL11B gene expression quantified using real-time quantitative PCR (qPCR) in cultured primary human AoSMCs was negligible (2^−ΔCt^: 0.003 ± 0.0005, *n* = 3) compared to that in PBMCs (724.4 ± 10.09, *n* = 3) or HEK293T cells (5.5 ± 0.16, *n* = 3, Fig. 2a), which have been reported in the literature to lack BCL11B expression.

### No evidence of BCL11B protein expression in the mouse aorta

The expression pattern of BCL11B was examined in wild-type 10-week-old mice, with the thymus, spleen and brain as positive controls (Supplementary Fig. 3A). Similar to the patterns observed in human tissues, BCL11B gene expression was detected in the aorta but at very low levels (2^−ΔCt^: 0.0003 ± 0.00001, *n* = 3) compared to the thymus (0.31 ± 0.006, *n* = 3) and spleen (0.029 ± 0.002, *n* = 3). BCL11B was below detection limits in western blots of aortic homogenates and IHC staining of FFPE sections of wild-type mice (Supplementary Fig. 3B-C). Similarly, BCL11B was below detection limits in western blots of aortic homogenates from BCL11B-tdTomato-expressing reporter mice using an anti-RFP antibody (Supplementary Fig. 4).

### BCL11B expression in the aorta reflects T cell infiltration

**Fig. 3:**
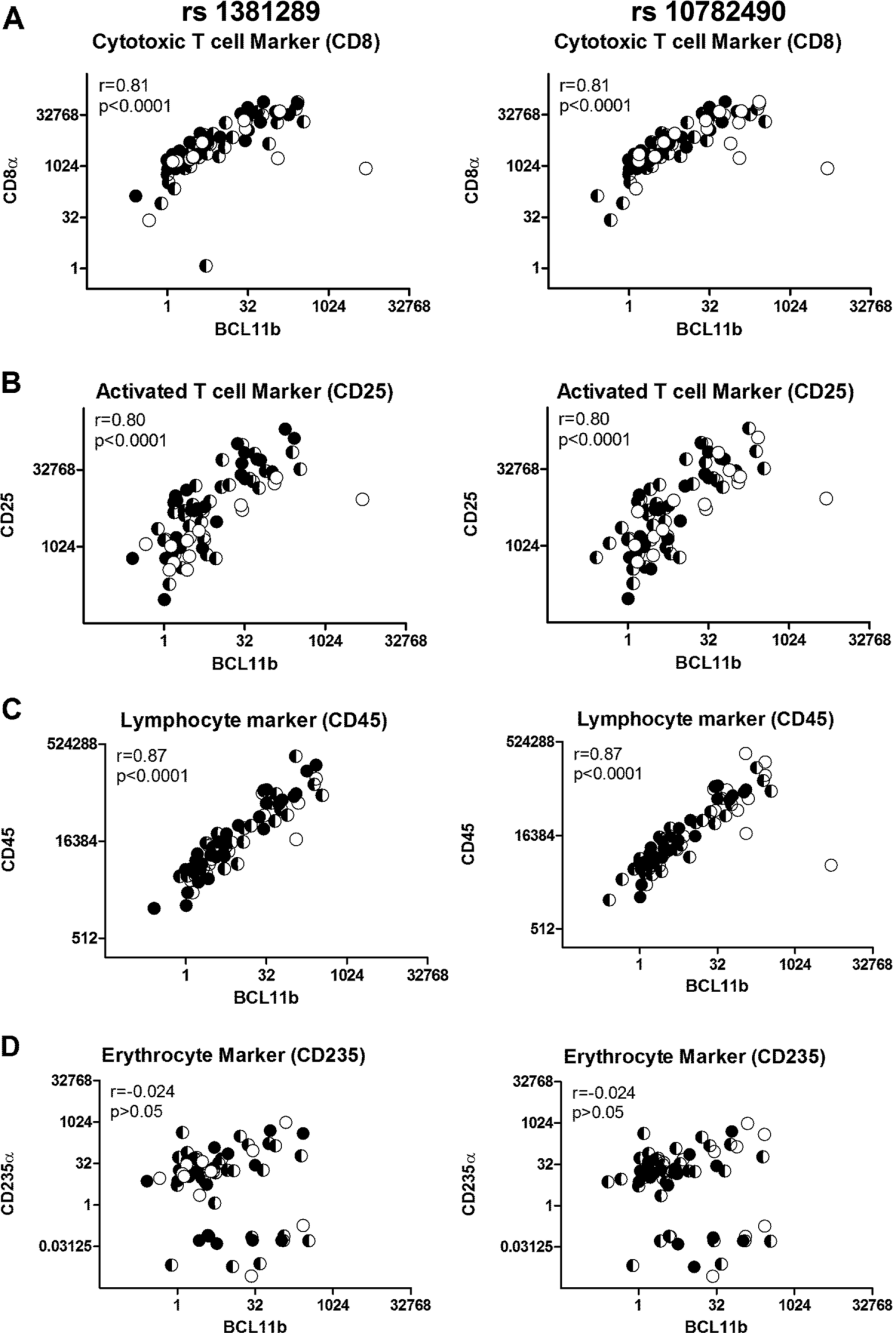
Aortic BCL11B expression correlates with leukocyte markers. Plots of BCL11B mRNA against and CD8α (**a**), CD25 (**b**), and CD45 (**c**) transcript levels (*n* = 85) showing a strong positive correlation between BCL11B and these leukocyte markers. There is no correlation with the erythrocyte marker CD235α (**d**). For both SNPs, the genotypes are shown as filled symbols (CC black, CT black/white, and TT white)

Since lymphocytes express BCL11B at high levels, we explored the possibility that aortic BCL11B gene expression reflected lymphocyte infiltration. We examined whether BCL11B transcript levels correlated with specific lymphocyte markers in randomly selected aortic samples (*n* = 85, Fig. 3a–c). We quantified the transcript levels of leukocyte common antigen CD45, the cytotoxic T-lymphocyte marker CD8α, and the activated T-lymphocyte marker CD25. BCL11B transcript correlated strongly with CD45 (*r* = 0.87, *P* < 0.0001), CD8α (*r* = 0.81, *P* < 0.0001), and CD25 (*r* = 0.80, *P* < 0.0001). This interpretation was further supported by IHC staining for CD45 in the aorta that showed higher levels of staining in the aortas with high BCL11B mRNA expression (Supplementary Fig. 5). We also considered whether these positive associations were due to inadequate removal of residual blood from the tissue samples by quantifying the expression of the specific erythrocyte marker CD235α. However, BCL11B gene expression did not correlate with CD235α gene expression (*r* = −0.02, *P* > 0.05, Fig. 3d).

## Discussion

Robust associations of genetic variants with cfPWV were identified in the most recent GWAS meta-analysis of >20,000 individuals. As with most complex traits, attaching biological function to these variants remains a challenge, especially when the risk locus lies in a gene desert. A growing body of evidence supports tissue-specific, long-range regulatory mechanisms for GWAS-identified risk SNPs [18–20]. The region of cfPWV association harbors gene-enhancer elements, such as DNAse-I hypersensitive sites, transcription factor-binding sites, and signatures of chromatin modification [5], and the region is able to drive T cell-specific BCL11B expression in a reporter system [6]. Hence, we examined whether the GWAS SNPs could drive BCL11B transcription. Using our human aortic resource, we found evidence that rs1381289 and rs10782490 are e-SNPs as BCL11B mRNA levels were significantly influenced by the genotype of these SNPs. Our data support BCL11B as a potential causal gene and suggest an inverse relationship between BCL11B transcript levels and PWV.

We provide further evidence of the biological importance of the 14q32.2 locus by demonstrating associations for two of the lead SNPs from the GWAS meta-analysis, rs1381289 and rs10782490, with PWV measured ex vivo in the proximal aorta. It is important to distinguish the molecular contributors of AS in the elastin-rich proximal aorta from the embryologically distinct and collagen-rich distal aorta. This is highlighted by regional differences in the BCL11B expression along the arterial tree (Supplementary Fig. 1), being higher in the ascending and thoracic aorta compared to the distal abdominal aorta and iliac arteries. An understanding of how these genetic variations influence AS is key for our better understanding of the molecular mechanisms that modify cfPWV.

Despite strong evidence for its association with AS, we found no evidence of BCL11B protein expression in our adult human aortas. Likewise, we did not detect BCL11B mRNA in cultured AoSMCs using the same qPCR assay. In view of this puzzling finding, and given that BCL11B is highly expressed in human T lymphocytes, we questioned whether it is constitutively expressed in the aorta, perhaps rapidly switched off in AoSMCs in culture, or whether what we detected is simply transcript abundance  from infiltrating lymphocytes. In fact, BCL11B gene expression correlated strongly with markers for total leukocytes and both activated and cytotoxic T lymphocytes, implying that immune mechanisms may underlie the association of BCL11B with AS. Lymphocytes have an important role in hypertension and the pathophysiological changes that result in the vessel wall [21]. Loss of BCL11B signaling can also have a proinflammatory stimulus. For example, the colons of mice lacking BCL11B display significant levels of infiltrated proinflammatory T helper type 1 (Th1) and Th17 CD4^+^ T cells, neutrophils and macrophages and develop inflammatory bowel disease [22]. A similar, BCL11B-dependent perturbation of the inflammatory system and inflammasome has been reported in other cell types. For example, inflammatory cell infiltration has been reported in the mouse epidermis where selective silencing of BCL11B in keratinocytes increased the infiltration of eosinophils, monocytes, CD3^+^ and CD4^+^ T lymphocytes, and CD45^+^ leukocytes in the skin [23]. Overall, the evidence indicates that BCL11B regulates a number of elements in the inflammatory pathway. And we would speculate that differential BCL11B expression among the SNP haplotypes modulates the lymphocyte infiltration signal we have identified.

An obvious question from our study is how a high BCL11B transcript abundance can be protective (associating inversely with AS) if the expression of BCL11B is proinflammatory. The aortas in our subjects clearly do not show histological evidence of inflammation in the sense of neutrophil or macrophage invasion. Recent work on lymphocyte subpopulations that are regulated by BCL11B are relevant here. Studies on type-2 innate lymphoid cells (ICL2) have shown them to have a key protective role in atherosclerosis [24]. In fact, mice genetically depleted of ICL2 lymphocytes show increased atherosclerosis [24]. The behavior of mature ICL2 lymphocytes is also tightly controlled by BCL11B including their eventual cytokine profile [25]. This suggests that ICL2s may be protective in the vessel wall, which clearly needs further exploration. It may be that peripheral ICL2 burden is predictive of PWV. If this were the case, it opens the way to possible therapeutic modulation of ICL2 function with exogenous Il-2 [24]. It also suggests a testable mouse model, as mice genetically depleted of ICL2 lymphocytes may have stiffer aortas than mice with replete ICL2 lymphocyte populations.

BCL11B may not be the only effector gene controlled by the 14q32.2 locus. The GWAS signal may modulate AS as part of a larger interplay of transcriptional regulation involving other targets flanking the 14q32.2 gene desert. Human vaccinia-related kinase 1 (VRK1) for instance lies ~ 1.1 MB centromeric to the locus. This prominent serine–threonine kinase in the nucleus complexes and promotes the stabilization and accumulation of p53 [26, 27]. Importantly, P53 is a transcription factor that tightly regulated by the transforming growth factor-β signaling cascade, itself a common pathway that underlies inherited aortopathies such as Marfan and Loeys–Dietz syndromes [28, 29]. Finally, p53 may itself be a target of BCL11B transcriptional control [30], so independent effects on VKR1 and BCL11B transcription potentially converge and potentiate AS. Close to the locus is the primate-specific, long non-coding RNA (lncRNA), DB129663. The enhancer region maps to the DB129663 promoter, and it is hypothesized that it is a target of the enhancer elements of the 14q32.2 gene desert [5]. LncRNAs are emerging as key players in transcriptional regulation, posttranscriptional modification, and epigenetic modulation and may therefore be central to the association of this region with AS [31]. The functional characteristics and expression patterns of DB129663, however, are currently unknown.

In conclusion, this is the first study investigating the role of the BCL11B gene desert SNPs on BCL11B expression and PWV measured ex vivo in a large sample of human aortas. We were unable to detect BCL11B at the protein level but provide evidence that suggests that the BCL11B transcript detected in the human aorta reflects lymphocyte infiltration. So we hypothesize that it is immune mechanisms that largely govern the association of BCL11B with AS. Further work incorporating other putative targets of the 14q32.2 enhancer elements may delineate the precise molecular mechanisms by which this locus can affect the stiffening process.

## Electronic supplementary material

Supplementary Data File
