# Supplementary material for: Functional characterization of common BCL11B gene desert variants suggests a lymphocyte-mediated association of BCL11B with aortic stiffness
Source: Eur J Hum Genet. 2018 Aug 8;26(11):1648–57. doi: 10.1038/s41431-018-0226-z (PMC6189060; doi:10.1038/s41431-018-0226-z)
Supplement: Supplementary file 1 — Supplementary Data File [file 41431_2018_226_MOESM1_ESM.pdf]

Supplementary File

Functional Characterization of Common BCL11B Gene Desert Variants suggests an Inflammation-Mediated Association of BCL11B with Aortic Stiffness, Al Maskari et al. 2018.

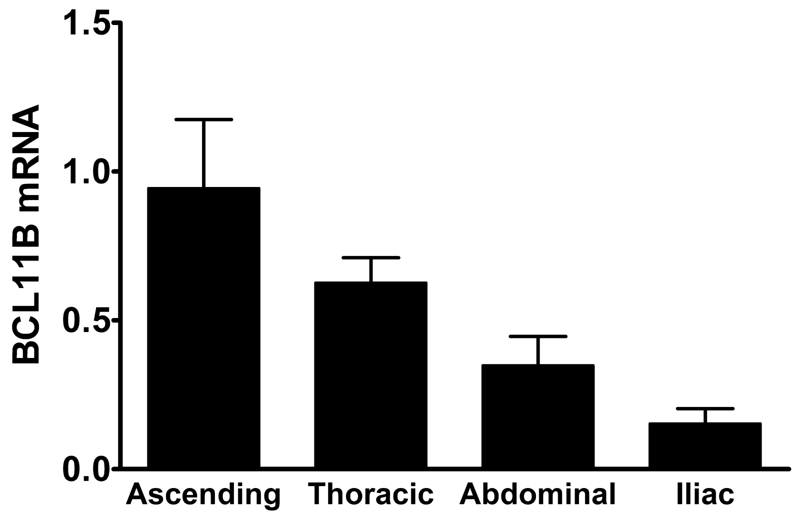

Supplementary Figure 1. Expression of BCL11B mRNA in different segments of the human aorta. Levels are the $2^{-\Delta Ct}$ values using GAPDH as house-keeper (mean ± SEM).

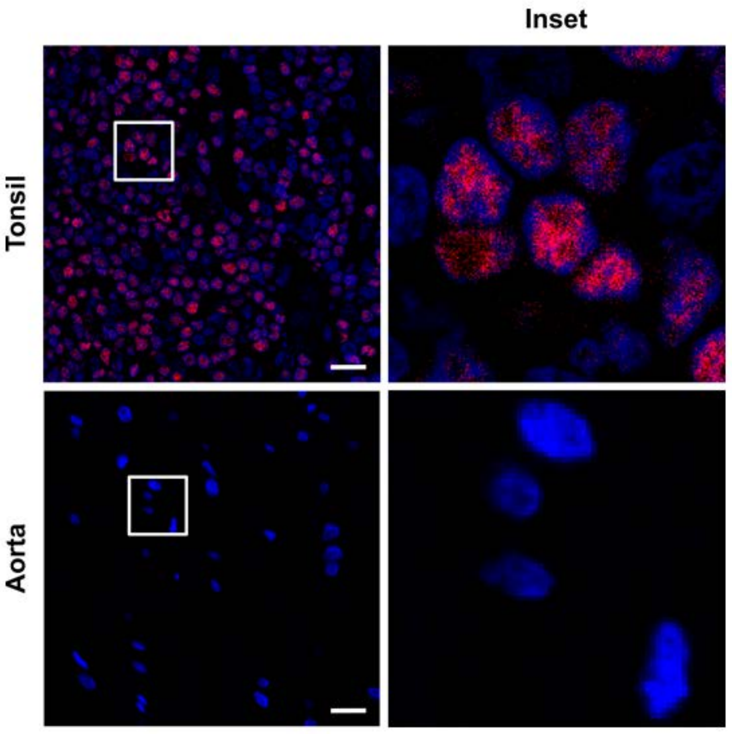

Supplementary Figure 2. Immunofluorescence staining for BCL11B with Tyramide Signal Amplification (TSA) in sections of human tonsil and aorta. The aortic section is representative of staining in 3 aortas which all showed high BCL11B mRNA expression.

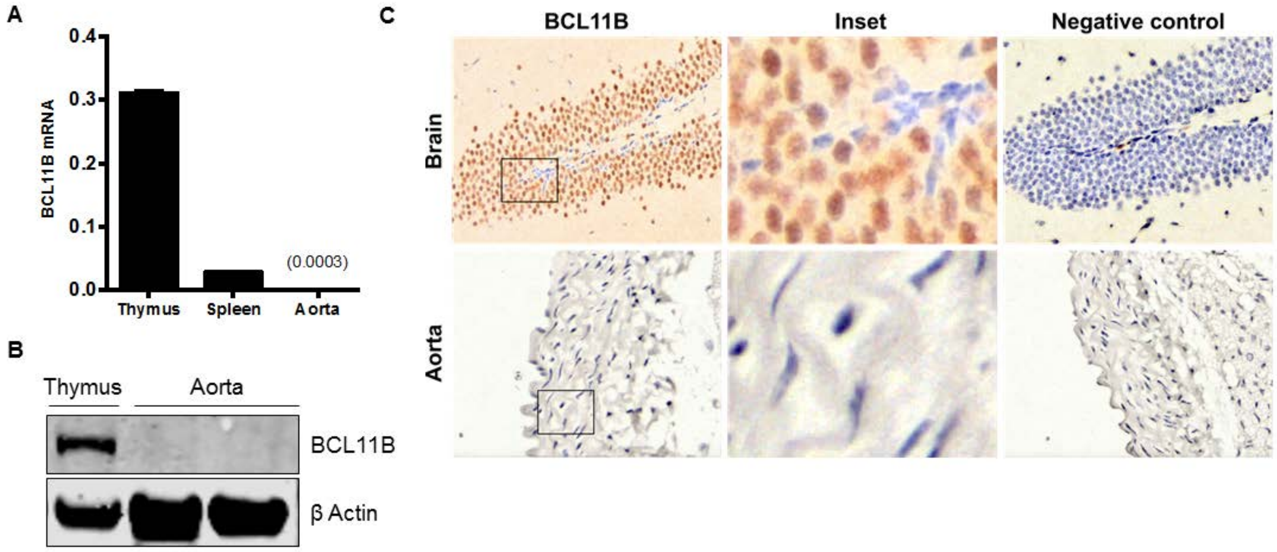

Supplementary Figure 3. Expression of BCL11B in 10 week-old mice. A. BCL11B mRNA quantified by qPCR (expressed as the $2^{-\Delta t}$ versus r18S). **B**. Immunoblot of aortic and thymus protein from the same mice. **C.** IHC staining for BCL11B using brain (hippocampus sections) as the positive control. Blots and staining are representative of results from 3 mice.

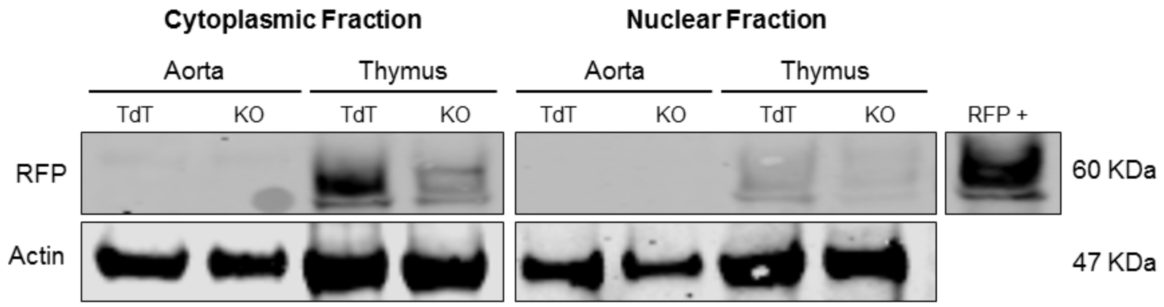

Supplementary Figure 4. Expression of BCL11B/tdTomato reporter construct in 10 week-old mice. The aortic and thymus protein extracts were fractionated before immunoblotting to show the relative distribution between the nucleus and cytosol. TdT= BCL11B-tdTomato reporter mice ($^{Bcl11bTd/flox}$)[38], KO= tdTomato reporter mice treated with tamoxifen (that conditionally knocks out the tdTomato cassette and exon 4 of BCL11b[38]). RFP+ is the positive control of a tdTomato-expressing human colorectal cancer cells.

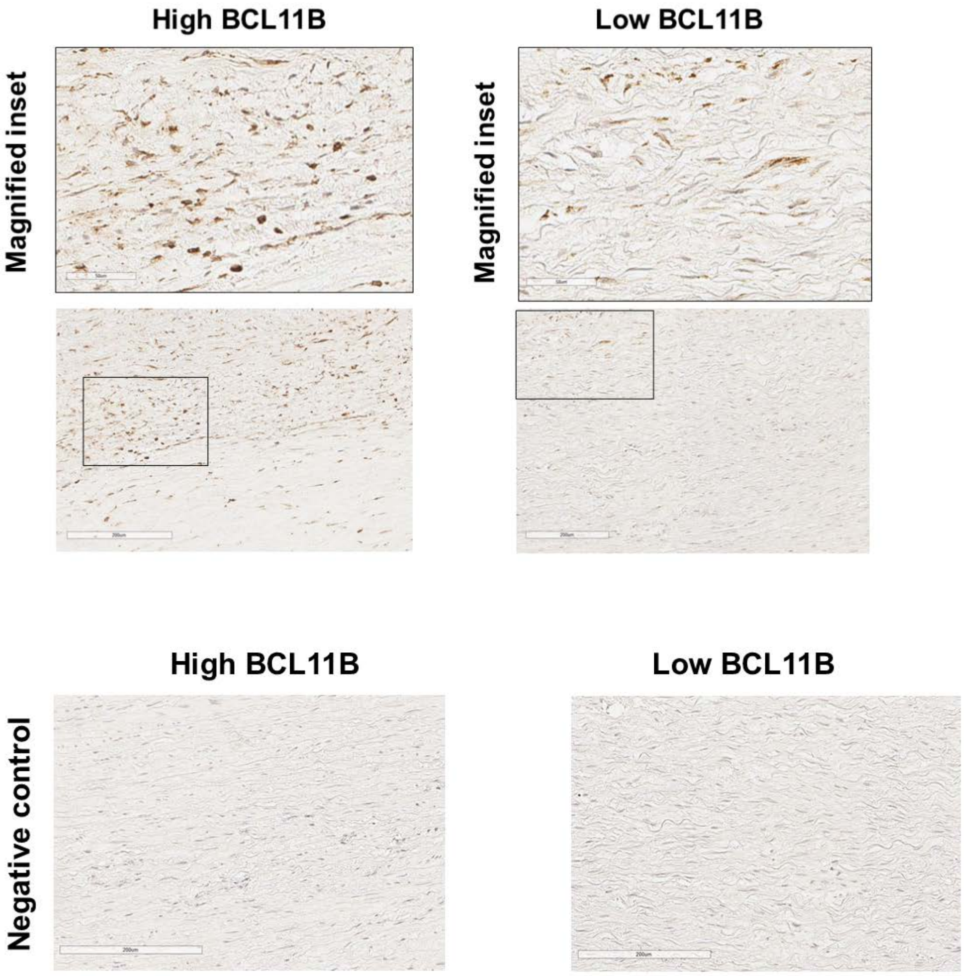

Supplementary Figure 5. Representative IHC staining for CD45 antigen on human aortic sections expressing either high or low levels of BCL11B mRNA.

Supplementary Table. Summarised phenotypic data by SNP genotype data.

Parameters	rs1381289 Genotypes				rs10782490 Genotypes				rs17773233 Genotypes			
	**CC**	**CT**	**TT**	**p-value**	**CC**	**CT**	**TT**	**p-value**	**GG**	**GT**	**TT**	**p-value**
Age (years)	58±14	54 ±16	55±15	0.333	56±15	54 ±16	58 ±15	0.217	56±15	53 ±17	64±10	0.124
Male/Female (n)	32/27	61/41	23/17	-	24/24	67/39	24/22	-	76/57	39/23	2/6	-
Height (m)	1.69±0.1	1.72±0.1	1.68±0.1	0.170	1.68±0.1	1.72±0.1	1.68±0.1	0.018	1.70±0.1	1.71±0.1	1.66±0.1	0.401
Weight (kg)	79.7±14	81.0±17	79.7±18	0.859	79.5±15	81.5±17	78.1±17	0.490	79.8±15	83.0±19	68.4±13	0.048
BMI (kg/m^2)	28.5±7.2	27.2 ±5.0	28.1±5.3	0.406	28.9±7.4	27.3±5.2	27.7±5.3	0.320	27.9±5.9	27.9±5.4	24.7±4.4	0.298
SBP (mmHg)	124±28	129 ±30	119±25	0.144	125±29	127±30	122±25	0.534	126±30	126±26	119±16	0.799
DBP (mmHg)	67±13	69±14	66±14	0.539	67±13	68±14	66±14	0.729	68±14	67±14	74±13	0.339
EM (MPa)	0.16 ±0.1	0.17±0.1	0.19±0.1	0.520	0.16±0.1	0.17±0.1	0.19±0.1	0.506	0.16±0.1	0.18±0.1	0.23±0.2	0.152
PWV (m/s)	3.78±0.8	3.90±1.1	4.00±1.1	0.585	3.77±0.8	3.87±1.1	4.05±1.1	0.428	3.80±0.9	4.05±1.3	4.00±0.9	0.275

Data are mean ± standard deviation.

BMI-body mass index, SBP-systolic blood pressure, DBP-diastolic blood pressure, EM-elastic modulus, PWV-pulse wave velocity.
